# Total Synthesis of Natural Disaccharide Sambubiose

**DOI:** 10.3390/ph13080198

**Published:** 2020-08-17

**Authors:** Simone Lucarini, Maria Gessica Ciulla, Paola Mestichelli, Andrea Duranti

**Affiliations:** Department of Biomolecular Sciences, University of Urbino Carlo Bo Piazza del Rinascimento, 6, 61029 Urbino, Italy; m.ciulla@campus.uniurb.it (M.G.C.); p.mestichelli@campus.uniurb.it (P.M.)

**Keywords:** chemopreventive phytochemicals, flavonoids, 2-*O*-xylosylvitexin, antiproliferative activity, carbohydrates, glycosylation, sambubiose, total synthesis

## Abstract

A practical and robust synthetic method to obtain the natural disaccharide sambubiose (2-*O*-β-D-xylopyranosyl-D-glucopyranose) is reported, exploring the key step in the synthesis, i.e., stereoselective *O*-glycosylation. Specifically, the best combinations of glycoside donors and acceptors were identified, stereospecific control of the reaction was achieved by screening several catalysts and protection/deprotection steps were evaluated and improved. The best result was obtained by coupling allyl 3,5,6-tri-*O*-benzyl-β-D-glucofuranoside with 2,3,4-tri-*O*-acetyl-D-xylopiranosyl-α-trichloro acetimidate in the presence of trimethylsilyl triflate as a catalyst giving the corresponding protected target compound as a correct single isomer. The latter was transformed accordingly into the desired final product by deprotection steps (deallylation, deacetylation, and debenzylation). Sambubiose was synthesized into a satisfactory and higher overall yield than previously reported and was also characterized.

## 1. Introduction

**Figure 1:**
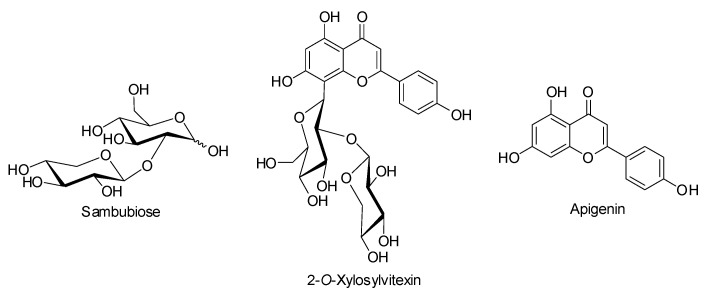
Chemical structures of sambubiose, 2-*O*-xylosylvitexin, and apigenin.

Chemopreventive phytochemicals (CPs) occur naturally in some plants and have been shown to inhibit all levels of carcinogenesis in both in vitro and in vivo models [1]. Among CPs, flavonoid 2-*O*-xylosylvitexin (Figure 1), which is constituted by carbohydrate (sambubiose, Figure 1) and aglycone (apigenin, Figure 1) components, was identified for the first time in *Citrus sinensis* leaves [2] and then in *Beta vulgaris* sub. *cycla* leaves and seeds [3] with the latter containing the most abundant and efficacious CPs.

2-*O*-xylosylvitexin shows strong antiproliferative activity against human colon cell cancer lines while also enhancing apoptosis, increasing cells in the G1 phase and reducing cells in the S phase as well as the proliferation of human fibroblasts [4].

Sambubiose, on the other hand, is formed by glucopyranose and xylopyranose units linked through a 1,2-trans bond in 2-position and was first isolated as a component of anthocyanins from elderberry (*Sambucus nigra*) [5], a plant with therapeutic properties [6,7]. In addition, sambubiose is also a constituent of the other major anthocyanins from black raspberries (*Rubus occidentalis* L.) [8], red currants, Davidson’s plums, and small red beans, and is linked to minor anthocyanins from açai and *Vaccinium padifolium* blueberries [9].

**Scheme 1:**
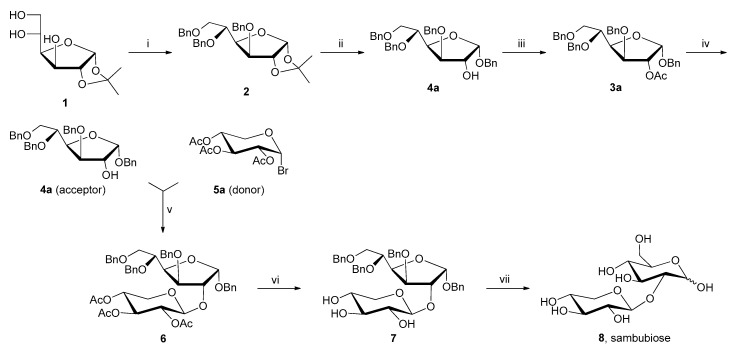
Reagents and conditions: (**i**) NaOH, BnCl, reflux, 7 h, 78%; (**ii**, **iii**) BnOH, HCl, reflux, 4 h then Ac_2_O, pyridine, rt, 24 h, 6%; (**iv**) MeONa, MeOH, rt, 12 h, 22%; (**v**, **vi**) Hg(CN)_2_/HgBr_2_, MeCN, rt, 4 h then MeONa, MeOH, rt, 12 h, 54%; (**vii**) H_2_, Pd/C 10%, EtOH, rt, 22 h, 43%.

To date, to the best of our knowledge, only one synthesis of sambubiose has been reported [10]; however, the method followed by Erbing and Lindberg [10] (Scheme 1) and subsequently applied by Dick et al. [11] has several drawbacks and improvements could be made in the reagents and conditions.

Firstly, the purification of benzyl 3,5,6-tri-*O*-benzyl-α-D-glucofuranoside (**4a**) to eliminate high-boiling benzyl alcohol from the reaction crude mixture was extremely difficult both by distillation and/or by chromatography. Erbing and Lindberg [10], therefore, had to employ acetylation and deacetylation as additional synthetic steps via benzyl 2-*O*-acetyl-3,5,6-tri-*O*-benzyl-α-D-glucofuranoside (**3a**) to purify **4a** (Scheme 1, “iii” and “iv” steps). Secondly, the use of the intrinsically unstable donor 2,3,4-tri-*O*-acetyl-α-D-xylopyranosyl bromide (**5a**), which must be freshly prepared and recrystallized because of its low stability, even when refrigerated, was also problematic. Moreover, Erbing and Lindberg [10] used a stoichiometric amount of mercuric cyanide as a glycosylation promoter. This reactant should not be used because of its high toxicity and dangerousness. Furthermore, Hg(CN)_2_ does not allow a stereospecific control of glycosylation: **6** [12] must be separated from its anomer since the output of the reaction is an α- and β-mixture. Finally, and importantly, the authors did not fully characterize the natural disaccharide sambubiose [10]. Hence, the aim of this investigation was to develop an improved efficient strategy to synthesize our target molecule overcoming the abovementioned drawbacks. The key synthetic step (i.e., the *O*-glycosylation between the appropriate protected glucose and activated/protected xylose) must be thoroughly explored. In particular, the leaving group of the donor should be screened, and the correct catalyst/promoter should be fine-tuned.

Herein, as a part of our ongoing investigations of disaccharide chemistry [13,14,15,16], we describe an efficient synthetic method that allowed us to obtain sambubiose in a satisfactory and higher overall yield than previously reported [10] as well as to achieve its full characterization. The proposed methodology ensured an effective stereospecific glycosylation control and exploited strategies of protection and deprotection of functional groups which also guide the regioselective reaction progress.

## 2. Results and Discussion

**Figure 2:**
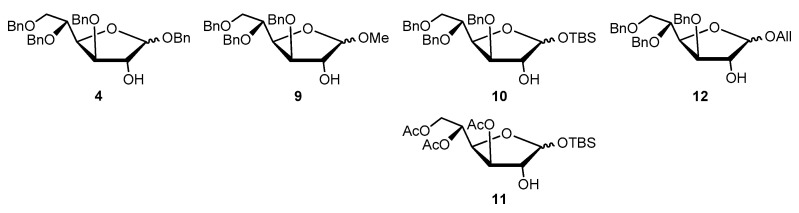
Chemical structure of synthesized glycoside acceptors.

The study started with the optimization of the key step, consisting of the *O*-glycosylation reaction between the appropriately protected glucose (acceptor) and protected/activated xylose (donor) (Scheme 1, step “v”). In particular, our attention was first focused on the synthesis of several glucose acceptors and xylose donors to overcome the abovementioned problems [10]. With regard to the glucose acceptor, the synthesis of compounds **9**–**12** (Figure 2) was designed and explored aiming to avoid the acetylation and deacetylation steps necessary for the purification of **4a** (Scheme 1).

**Scheme 2:**
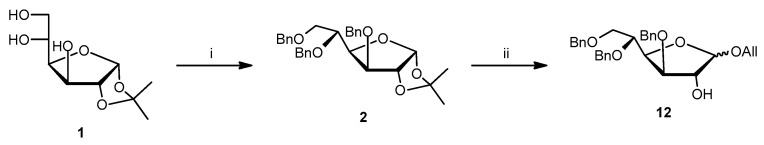
Reagents and conditions: (i) 80% NaH, BnBr, DMF, rt, 6 h, 70%; (ii) AllOH, TsOH, reflux, 3 h, 85%.

Methyl 3,5,6-tri-*O*-benzyl-D-glucofuranoside (**9**) is very easy to purify and relatively stable under basic or acid conditions. Compound **9** was synthesized starting from 3,5,6-tri-*O*-benzyl-1,2-*O*-isopropylidene-D-glucofuranoside (**2**, Scheme 2) (1% HCl, MeOH, 65 °C, 1.5 h, 91%; α/β 1:1.3), previously obtained by 1,2-*O*-isopropylidene-D-glucofuranoside (**1**, Scheme 2).

Likewise, *tert*-butyldimethylsilyl 3,5,6-tri-*O*-benzyl-D-glucofuranoside (**10**, Figure 2) was synthesized from **2** via a vicinal diol intermediate. Unfortunately, **10** was obtained with a very low overall yield (1,4-dioxane, H_2_SO_4_, reflux, 3 h, 41%; TBSCl, imidazole, THF, 0 °C, 10 h, 10%; α/β 1:1). On the other hand, the attempt to change the “armed” protective groups with the “disarmed” *tert*-butyldimethylsilyl 3,5,6-tri-*O*-acetyl-D-glucofuranoside (**11**, Figure 2) also proved not to be effective in term of yields (Ac_2_O, Py, rt, 17 h, 85%; TFA, 50 °C, 3 h, 19%; TBSCl, imidazole, THF, 0 °C, 10 h; 10%). Both **10** and **11** were not assessed as acceptors in the glycosylation reaction. Finally, our attention was turned to the glycoside acceptor allyl 3,5,6-tri-*O*-benzyl-D-glucofuranoside (**12**, Figure 2) which was prepared in an acidic environment by treating **2** with allyl alcohol [17] giving an α/β 1:1.1 mixture (Scheme 2).

**Scheme 3:**
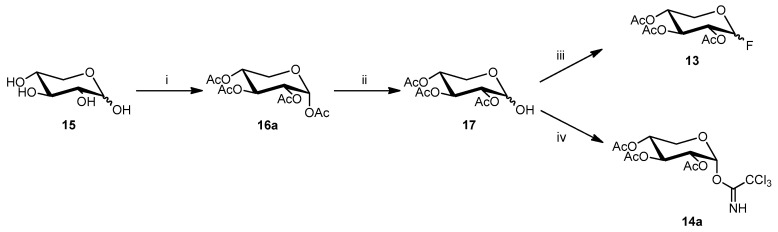
Reagents and conditions: (**i**) Ac_2_O, Py, 0 °C, 6 h, 75%; (**ii**) NH_4_OAc, DMF, rt, 22 h, 70%; (**iii**) DAST, CH_2_Cl_2_, −30 °C to rt, 1 h, 98%; (**iv**) CCl_3_CN, CH_2_Cl_2_, 0 °C, DBU, 1 h, 60%.

With regard to the donor glycoside, only compounds that are more stable than **5a** (Scheme 1), such as fluoride [18] and trichloroacetimidate [19] derivatives, two of the most appealing and promising glycoside donors for *O*-glycosylation, were taken into account. Hence, 2,3,4-tri-*O*-acetyl-α-D-xylopyranosyl fluoride (**13**, Scheme 3) and 2,3,4-tri-*O*-acetyl-α-D-xylopyranosyl trichloroacetimidate (**14a**, Scheme 3) were synthesized.

In the case of fluoride **13** [20], the desired hemiacetal intermediate 2,3,4-tri-*O*-acetyl-D-xylopyranoside (**17**, Scheme 3) was prepared starting from D-(+)-xylose (**15**) through an initial acetylation (Ac_2_O, Py, 0 °C, 6 h, 99%) followed by monohydrolysis of the corresponding D-xylopyranose 1,2,3,4-tetracetate (**16a**) [21] with ammonium acetate (DMF dry, rt, 22 h, 70%). Glycoside donor **13** was then obtained by fluorination of **17** with diethylaminosulfur trifluoride (DAST) (CH_2_Cl_2_, −30 °C to rt, 1 h, 98%) as a mixture of anomers (α/β 1:4), which could be easily isolated by a flash chromatography.

With regard to trichloroacetimidate **14a** [22], this donor type showed some advantages such as a relatively high stability, easy purification, and, most importantly, a potential stereochemical control of the glycosylation reaction. In addition, **14a** was synthesized by treating **17** with trichloroacetonitrile (CCl_3_CN) and 1,8-diazabicyclo [5.4.0]undec-7-ene (DBU) obtaining only α-anomer [23]. As previously reported, DBU promoted thermodynamic control of the reaction, which led to a more stable α-anomer **14a** (Scheme 3). One-pot attempts to obtain **14a** from **16a** were also explored; however, such attempts proved not to be advantageous due to the fact of their low yields (data not shown).

**Scheme 4:**
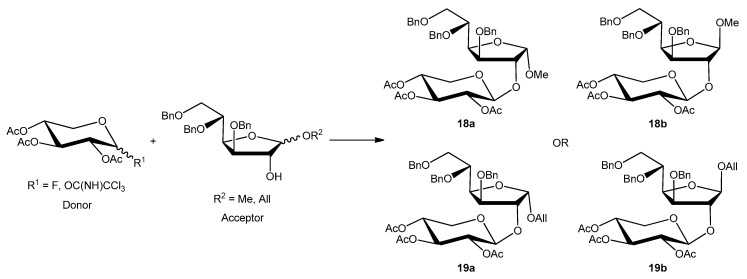
General scheme of *O*-glycosylation.

Finally, the key synthetic step, consisting in the *O*-glycosylation reaction, was explored. The initial substitution of mercuric salts [10] with silver oxide or silver carbonate as an activator was ineffective any advantage because the yields in these conditions fell drastically (data not shown). The use of safer, greener catalysts such as boron trifluoride diethyl etherate (BF_3_ **^.^** OEt_2_) or trimethylsilyl trifluoromethanesulfonate (TMSOTf, particularly suitable for the glycoside donor activation bearing the trichloroacetoimidate group as the leaving group), warrant further investigation as alternatives to mercury compounds. Dichloromethane was used as a non-polar solvent; therefore, the stereoselection of glycosylation was led by the 2-*O*-acyl vicinal group on the donor (anchimeric assistance). All the prepared glycoside donors and acceptors were reacted in different conditions in the presence of powdered molecular sieves (MS) 4Å (Scheme 4) at −20 °C, and the main results are reported in Table 1.

Firstly, the reactivity of fluoride glycoside **13b** was explored with the methoxy derivative **9b** or **9a** as a glycoside acceptor, in the presence of BF_3_^.^ OEt_2_ at −20 °C to room temperature for 4 h. The reaction gave the corresponding β- or α-disaccharide in 80% or 50% yields, respectively, and a complete *trans* stereocontrol (entries 1 and 2).

A complete recovery of the acceptor (**9b** or **9a**) was pointed out in both reactions. The lower yield for glycosylation obtained using the acetate **9a** could be attributed to the steric hindrance of the methoxy group present in the same part of the plane of the sugar of the reactive 2-OH group. Similarly, the allyloxy acceptor **12b** gave the corresponding coupling product in 30% yield, and its anomer **12a** did not react at all and was completely recovered (entries 6 and 3, respectively). The absence of reactivity of **12a** could be explained by the greater steric hindrance of its 1-allyloxy substituent compared to that of 1-methoxy present on **9a** (entry 3 versus 2). On the other hand, the α-anomer fluoride donor **13a** did not react with either highly reactive acceptor **9b** or **12b** in the same conditions (entries 4 and 5, respectively). The last donor to be investigated was trichloroacetimidate **14a**, which was reacted with **9b** (entry 7) in the presence of BF_3_^.^ OEt_2_, giving the corresponding β-linked disaccharide in 44% yield or with **12b** (entry 8) using TMSOTf as a promoter and obtaining a better yield (60%), complete stereocontrol, and high reproducibility. Surprisingly, the use of the latter conditions (donor **14a** and promoter TMSOTf) and very low reactive donor **12a** allowed coupling with the formation of the corresponding disaccharide; however, with a lower yield (45%) and longer reaction time than anomer **12b** (entry 9). Hence, with regard to *O*-glycosylation, the best overall result in terms of yield, stereocontrol, and reproducibility was obtained by reacting donor 14a and acceptor **12b** promoted by TMSOTf.

We then focused on the deprotection steps of 3,5,6-tri-*O*-benzyl-2-*O*-(2,3,4-tri-*O*-acetyl-β-D-xylopiranosyl)-D-glucofuranoside **18a** and **18b** or **19a** and **19b** (Scheme 4) with the aim of maximizing the efficiency of the synthetic strategy to obtain sambubiose.

*O*-Methoxy group deprotection in the disaccharides **18a** and **18b** were first investigated using strong acids such as hydrochloride acid (HCl, rt or −15 °C, 10 h) or BF_3_^.^ OEt_2_ (0 °C, 10 h). Unfortunately, demethylation was not obtained in any of these cases, and degradation of the starting material or no reaction was observed. Also treating disaccharides with trityl tetrafluoroborate [(C_6_H_5_)_3_C^.^BF_4_] [24] (rt or −15 °C, 4–12 h), usually employed in the deprotection of benzyl and methyl ethers on anomeric carbons, was found to be detrimental (data not shown).

Hence, **18b** was also first debenzylated by hydrogenolysis (10% Pd/C, EtOAc/EtOH 1:2, 32%) leading to the derivative β-methyl 2-*O*-(2,3,4-tri-*O*-acetyl-β-D-xylopyranosyl)-D-glucofuranoside (**20b**) which, in turn, was deacetylated (NaOMe, MeOH, 7 h, 70%) to give β-methyl 2-*O*-β-D-xylopyranosyl-D-glucofuranoside (**21b**). Attempts to obtain sambubiose by deprotection of **20b** and **21b** by cleaving the methoxy group also failed (data not shown).

**Scheme 5:**
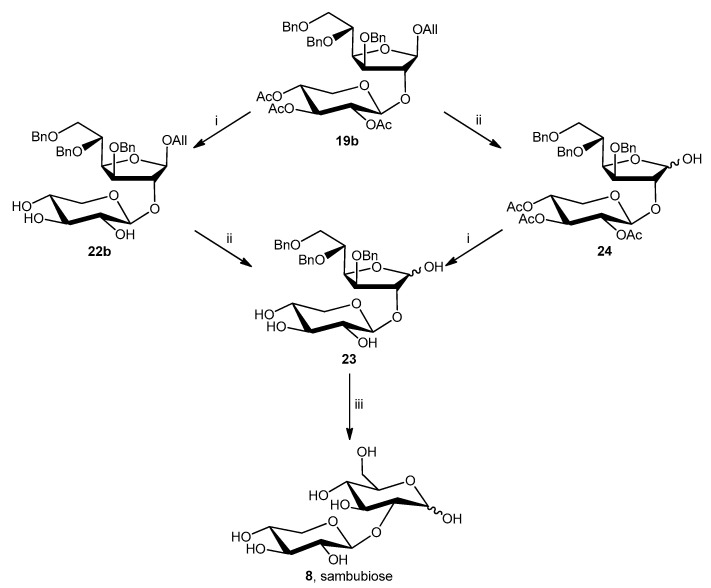
Reagents and conditions: (**i**) MeONa, MeOH, rt, 3 h, 90% (**22b**) or 38% (**23**); (**ii**) MeCOONa, MeCOOH, PdCl_2_, H_2_O, rt, 12 h, 42% (**23**) or 70% (**24**); (**iii**) H_2_, Pd/C 10%, EtOH, rt, 6 h, 51%.

In the case of allyloxy disaccharides **19a** and **19b**, sambubiose might theoretically be obtained by two different deprotection routes (compound **19b** is reported in Scheme 5 as an example), both of which were explored. The first, on the left in Scheme 5, envisaged *O*-deacetylation, *O*-deallylation, and, finally, *O*-debenzylation. On the other hand, the second route called for *O*-deallylation before *O*-deacetylation. The initial deprotection route involved *O*-deacetylation of compounds **19a** or **19b**. The compounds were treated using a typical condition for the hydrolysis of the acetyl esters with sodium methoxide and gave the corresponding desired compounds α- and β-allyl 3,5,6-tri-*O*-benzyl-2-*O*-β-D-xylopyranosyl-D-glucofuranoside in good yield (**22a**, 70%; **22b**, 90%). Subsequently, the deprotection of the allyl group was investigated with **22a** and **22b**. Several methods were explored, including palladium chloride [25] (MeCOONa, MeCOOH, PdCl_2_, H_2_O, rt, 12 h, 42%), Wilkinson’s catalyst [26] [(Ph_3_P)_3_RhCl, toluene:MeOH, rt to reflux, 20 h, 39%] or hydrogen activated iridium complex [27] {[Ir(COD)(PCH_3_Ph_2_)_2_]PF_6_, H_2_, TsOH, CH_2_Cl_2_/MeOH, 19%}, but only β-anomer **22b** reacted, probably due to the steric hindrance of α-one. Therefore, **22b** was the only possible anomer suitable for the synthesis of **23** (3,5,6-tri-*O*-benzyl-2-*O*-β-D-xylopyranosyl-α-D-glucofuranose and 3,5,6-tri-*O*-benzyl-2-*O*-β-D-xylopyranosyl-β-D-glucofuranose) which was obtained as an anomeric mixture.

On the other hand, using the second approach (on the right in Scheme 5), **19a** and **19b** were deallylated with palladium chloride or Wilkinson’s catalyst under the previously described conditions. Disaccharide **19b** gave the corresponding product 3,5,6-tri-*O*-benzyl-2-*O*-(2,3,4-tri-*O*-acetyl-β-D-xylopiranosyl)-D-glucofuranose (**24**) in good yields with both methods. However, compound 19a was deprotected only by (Ph_3_P)_3_RhCl) in low yield (23%). Then, **24** was deacetylated using sodium methoxide and methanol to give **23**. In conclusion, the deprotection scheme on the left used to obtain **23** was more effective in terms of overall yield than the one on the right (38% versus 27%). Finally, sambubiose was obtained by *O-*debenzylation of **23** which was treated with palladium under hydrogen atmosphere. During the last deprotection, the five-membered ring (glucofuranose) of compound **23** spontaneously transformed into a six-membered ring (glucopyranose).

**Scheme 6:**
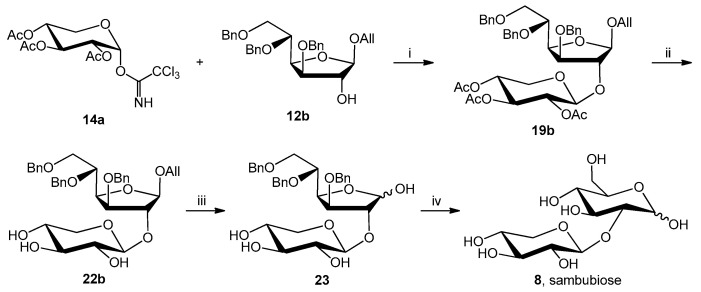
Reagents and conditions: (**i**) TMSOTf, CH_2_Cl_2_, –20 °C to rt, 3.5 h, 60%; (**ii**) MeONa, MeOH, rt, 3 h, 90%; (**iii**) MeCOONa, MeCOOH, PdCl_2_, H_2_O, rt, 12 h, 42%; (**iv**) H_2_, Pd/C 10%, EtOH, rt, 6 h, 51%.

The final four improved/developed steps are reported in Scheme 6.

Interestingly, sambubiose was obtained with a satisfactory overall yield (7%) starting from the acceptor precursor **1** compared to the 0.24% yield reported in the literature [10].

## 3. Materials and Methods 

### Chemicals, Materials, and Methods

All reagents were purchased from Sigma–Aldrich (St. Louis, MO, USA), Alfa Aesar (Haverhill, MA, USA), or TCI (Tokyo, Japan) in the highest quality commercially available. The solvents were RP grade. The melting points were determined using the Büchi (Flawil, Switzerland) B-540 apparatus. The purity of the products was evaluated unequivocally through MS, ^1^H NMR, ^13^C NMR, IR, and [α]^20^_D_. The MS (ESI) spectra were recorded with a Waters (Milford, MA, USA) Micromass ZQ spectrometer in a positive mode using a nebulizing nitrogen gas at 400 L/min and a temperature of 250 °C, cone flow 40 mL/min, capillary 3.5 K volts, and cone voltage 60 V; the revelation was performed either in ESI (+) and/or ESI (−), from 100 to 800 units of mass, spectrophotometrically measured using a diode array spectrophotometer. The ^1^H NMR and ^13^C NMR spectra were recorded on a Bruker (‎Billerica, MA‎, USA) AC 400 or 101 instrument and analyzed using the Top Spin 1.3 (2013) Bruker NMR software package. Chemical shifts were measured using the central peak of the solvent. Purification of the crude material was carried out by column chromatography on silica gel “flash” [230–400 mm, Merck (Darmstadt‎, Germany)]. The TLC analyses were performed on pre-coated aluminum oxide on aluminum sheets (60 F254, neutral; Merck). The IR spectra were obtained with a Thermo Fisher Scientific (Waltham, MA‎, USA) Nicolet Avatar 360 spectrophotometer. The optical rotations were measured on the Digital Perkin Elmer (Waltham, MA‎, USA) 241 polarimeter using a sodium lamp (589 nm) as a light source; concentrations were expressed in g/100 mL, and the length of the cell was 1 dm.

*Synthesis of allyl 3,5,6-tri-O-benzyl-α-D-glucofuranoside (**12a**) and allyl 3,5,6-tri-O-benzyl-β-D-glucofuranoside* (**12b**). To a solution of **2** (1 g, 2.04 mmol) [obtained by treating **1** (2 g, 9.08 mmol) with 80% NaH (0.9 g, 30 mmol), BnBr (4.66 g, 27.25 mmol) in DMF (40 mL)] at room temperature for 6 h in allyl alcohol (14.6 mL) and under stirring, TsOH (0.062 g, 0.33 mmol) was added. After the reactants were refluxed for 3 h, the mixture was cooled, extracted with CH_2_Cl_2_, washed with NaHCO_3_ sat., dried over Na_2_SO_4_, filtered and concentrated. Purification of the residue by column chromatography (cyclohexane/EtOAc 9:1) gave **12a** and **12b** as light yellow oils, which were dried under N_2_ atmosphere because of their thermolability (*R*_f_: **12a** 0.4, **12b** 0.3; cyclohexane/EtOAc 6:4). Yield: **12a** 40% (0.4 g, 0.82 mmol); **12b** 45% (0.45 g, 0.92 mmol). **12a**: MS (ESI): 490 (M + H^+^). ^1^H NMR (CDCl_3_) *δ*: 3.61 (dd, 1H, *J*_6b–5_ = 5.8 Hz, *J*_6b–6a_ = 10.6 Hz, H^6b^), 3.78 (dd, 1H, *J*_6a–5_ = 2.0 Hz, *J*_6a–6b_ = 10.6 Hz, H^6a^), 3.95 (ddd, 1H, *J*_5–6a_ = 2.0 Hz, *J*_5–6b_ = 5.8 Hz, *J*_5–4_ = 8.4 Hz, H^5^), 4.00 (dd, 1H, *J*_3–2_ = 1.9 Hz, *J*_3–4_ = 4.4 Hz, H^3^), 4.01 (dddd, 1H, *J*_7b–9a_ = *J*_7b–9b_ = 1.5 Hz, *J*_7b–8_ = 6.2 Hz, *J*_7b–7a_ = 12.8 Hz, H^7b^ OCH*H*CHCH_2_), 4.22 (dddd, 1H, *J*_7a–9a_ = *J*_7a–9b_ = 1.5 Hz, *J*_7a–8_ = 5.2 Hz, *J*_7a–7b_ = 12.8 Hz, H^7a^ OC*H*HCHCH_2_), 4.26 (dd, 1H, *J*_2–3_ = 1.9 Hz, *J*_2–1_ = 4.5 Hz, H^2^), 4.27 (dd, 1H, *J*_4–3_ = 4.4 Hz, *J*_4–5_ = 8.4 Hz, H^4^), 4.45 (d, 1H, *J* = 11.5 Hz, OC*H*_2_C_6_H_5_), 4.47 (d, 1H, *J* = 11.5 Hz, OC*H*_2_C_6_H_5_), 4.49 (d, 1H, *J* = 12.2 Hz, OC*H*_2_C_6_H_5_), 4.53 (d, 1H, *J* = 12.2 Hz, OC*H*_2_C_6_H_5_), 4.63 (d, 1H, *J* = 11.5 Hz, OC*H*_2_C_6_H_5_), 4.72 (d, 1H, *J* = 11.5 Hz, OC*H*_2_C_6_H_5_), 5.11 (d, 1H, *J*_1–2_ = 4.5 Hz, H^1^), 5.12 (dddd, 1H, *J*_9b–9a_ = *J*_9b–7a_ = *J*_9b–7b_ = 1.5 Hz, *J*_9b–8_ = 10.2 Hz, H^9b^ OCH_2_CHCH*H*), 5.19 (dddd, 1H, *J*_9a–9b_ = *J*_9a–7a_ = *J*_9a–7b_ = 1.5 Hz, *J*_9a–8_ = 17.2 Hz, H^9a^ OCH_2_CHC*H*H), 5.83 (dddd, 1H, *J*_8–7a_ = 5.2 Hz, *J*_8–7b_ = 6.2 Hz, *J*_8–b_ = 10.2 Hz, *J*_8–9a_ = 17.2 Hz, H^8^ OCH_2_C*H*CH_2_), 7.24–7.37 (m, 15H, Ar*H*) ppm. ^13^C NMR (CDCl_3_) *δ*: 69.2, 71.2, 71.7, 72.6, 73.4, 76.1, 77.9, 83.9, 100.4, 117.7, 127.5, 127.5, 127.6, 128.1, 128.2, 128.3, 133.7, 137.9, 138.6, 138.9 ppm. IR (Nujol) ν = 3550, 1454, 1372, 1206, 1057 cm^−1^. [α]^20^_D_ = –56.8 (c 0.3, CHCl_3_). **12b**: MS (ESI): 490 (M + H^+^). ^1^H NMR (CDCl_3_) *δ*: 3.63 (dd, 1H, *J*_6b–5_ = 5.3 Hz, *J*_6b–6a_ = 10.7 Hz, H^6b^), 3.80 (dd, 1H, *J*_6a–5_ = 2.0 Hz, *J*_6a–6b_ = 10.7 Hz, H^6a^), 3.90 (dddd, 1H, *J*_7b–9a_ = *J*_7b–9b_ = 1.5 Hz, *J*_7b–8_ = 6.0 Hz, *J*_7b–7a_ = 13.0 Hz, H^7b^ OCH*H*CHCH_2_), 4.00 (ddd, 1H, *J*_5–6a_ = 2.0 Hz, *J*_5–6b_ = 5.3 Hz, *J*_5–4_ = 8.8 Hz, H^5^), 4.00 (dd, 1H, *J*_3–2_ = 1.0 Hz, *J*_3–4_ = 5.0 Hz, H^3^), 4.14 (dddd, 1H, *J*_7a–9a_ = *J*_7a–9b_ = 1.5 Hz, *J*_7a–8_ = 5.0 Hz, *J*_7a–7b_ = 13.0 Hz, H^7a^ OC*H*HCHCH_2_), 4.21 (dd, 1H, *J*_2–1_ ≅ *J*_2–3_ = 1.0 Hz, H^2^), 4.33 (dd, 1H, *J*_4–3_ = 5.0 Hz, *J*_4–5_ = 8.8 Hz, H^4^), 4.43 (d, 1H, *J* = 11.5 Hz, OC*H*_2_C_6_H_5_), 4.47 (d, 1H, *J* = 11.5 Hz, OC*H*_2_C_6_H_5_), 4.52 (s, 2H, OC*H*_2_C_6_H_5_), 4.57 (d, 1H, *J* = 12.2 Hz, OC*H*_2_C_6_H_5_), 4.67 (d, 1H, *J* = 11.5 Hz, OC*H*_2_C_6_H_5_), 4.87 (d, 1H, *J*_1–2_ = 1.0 Hz, H^1^), 5.10 (dddd, 1H, *J*_9b–9a_ = *J*_9b–7a_ = *J*_9b–7b_ = 1.5 Hz, *J*_9b–8_ = 10.5 Hz, H^9b^ OCH_2_CHCH*H*), 5.22 (dddd, 1H, *J*_9a–9b_ = *J*_9a–7a_ = *J*_9a–7b_ = 1.5 Hz, *J*_9a–8_ = 17.0 Hz, H^9a^ OCH_2_CHC*H*H), 5.83 (dddd, 1H, *J*_8–7a_ = 5.0 Hz, *J*_8–7b_ = 6.0 Hz, *J*_8–9b_ = 10.5 Hz, *J*_8–9a_ = 17.0 Hz, H^8^ OCH_2_C*H*CH_2_), 7.24–7.37 (m, 15H, Ar*H*) ppm. ^13^C NMR (CDCl_3_) *δ*: 68.9, 70.8, 72.0, 72.5, 73.4, 76.7, 77.0, 77.3, 77.3, 78.6, 79.9, 83.0, 107.8, 117.0, 127.6, 127.7, 128.2, 128.3, 128.3, 134.2, 134.3, 138.0, 138.6, 138.9 ppm. IR (Nujol) ν = 3423, 1462, 1398, 1061 cm^−1^. [α]^20^_D_ = +19.5 (c 0.2, CHCl_3_). The physico-chemical data are in agreement with those reported in the literature [28].

*Synthesis of 1,2,3,4-tetra-O-acetyl-*α*-D-xylopyranose* (**16a**). To a solution of **15** (1 g, 6.66 mmol) in pyridine (5 g, 4.5 mL, 62 mmol), Ac_2_O was added (5.5 g, 4.5 mL, 52.74 mmol). The mixture was stirred at 0 °C for 6 h, extracted with Et_2_O and washed with H_2_O and CuSO_4_ saturated solution. The combined organic layers were dried with Na_2_SO_4_, filtered and concentrated. Purification of the residue by column chromatography (cyclohexane/EtOAc 9:1) gave **16a** as a yellow oil which was dried under N_2_ atmosphere because of its thermolability. Yield: 75% (1.59 g, 5 mmol). MS (ESI): 336 (M + NH_4_^+^). ^1^H NMR (CDCl_3_) *δ*: 2.04–2.19 (m, 12H, C*H*_3_CO), 3.72 (dd, 1H, *J*_5b–4_ = *J*_5b–5a_ = 11.0 Hz, H^5b^), 3.95 (dd, 1H, *J*_5a–4_ = 6.0 Hz, *J*_5a–5b_ = 11.0 Hz, H^5a^), 5.04 (dd, 1H, *J*_2–1_ = 3.5 Hz, *J*_2–3_ = 10.0 Hz, H^2^), 5.05 (ddd, 1H, *J*_4–5a_ = 6.0 Hz, *J*_4–3_ = 10.0 Hz, *J*_4–5b_ = 11.0 Hz, H^4^), 5.48 (dd, 1H, *J*_3–2_ = *J*_3–4_ = 10.0 Hz, H^3^), 6.27 (d, 1H, *J*_1–2_ = 3.5 Hz, H^1^) ppm. ^13^C NMR (CDCl_3_) *δ*: 20.4, 20.6, 20.7, 20.8, 60.6, 68.7, 69.3, 89.2, 168.9, 169.6, 169.7, 170.1 ppm. IR (film) ν = 1766, 1232, 1128 cm^−1^. [α]^20^_D_ = +88.0 (c 0.3, CHCl_3_). ^1^H NMR, ^13^C NMR, and IR data are in agreement with those reported in the literature [21].

*Synthesis of 2,3,4-tri-O-acetyl-D-xylopyranose* (**17**). To a solution of **16a** (1 g, 3.14 mmol) in dry DMF (9 mL), AcONH_4_ (0.485 g, 6.29 mmol) was added and the mixture was stirred at room temperature for 22 h, extracted with EtOAc, and washed with H_2_O and saturated aq. NH_4_Cl solution. The combined organic layers were dried with Na_2_SO_4_ and concentrated. Purification of the oily residue by column chromatography (cyclohexane/EtOAc 7:3) gave **17** as a white solid. Yield 70% (0.607 g, 2.2 mmol). Mp: 142–144 °C (EtOAc/petroleum ether). MS (ESI): 299 (M + Na^+^). [α]^20^_D_ = +69.0 (c 0.2, CHCl_3_). ^1^H NMR, ^13^C NMR, and IR data are in agreement with those reported in the literature [29].

*Synthesis of 2,3,4-tri-O-acetyl-D-xylopyranosyl-α-trichloroacetimidate* (**14a**). To a solution of **17** (0.407 g, 1.48 mmol) and CCl_3_CN (1.25 g, 0.86 mL, 8.83 mmol) in dry CH_2_Cl_2_ (14 mL) at 0 °C and under N_2_ atmosphere, DBU was added (0.09 g, 0.88 mL, 0.59 mmol) and the mixture was stirred at 0 °C for 1 h and then concentrated. Purification of the residue by column chromatography (cyclohexane/EtOAc 9:1) gave **14a** as a yellow oil (*R*_f_: 0.5, cyclohexane/EtOAc 7:3). Yield: 60% (0.37 g, 0.86 mmol). MS (ESI): 437 (M + NH_4_^+^). ^1^H NMR (CDCl_3_) *δ*: 2.03 (s, 3H, C*H*_3_CO), 2.06 (s, 3H, C*H*_3_CO), 2.07 (s, 3H, C*H*_3_CO), 3.82 (dd, 1H, *J*_5a–4_ = *J*_5a–5b_ = 11.0 Hz, H^5a^), 4.00 (dd, 1H, *J*_5b–4_ = 5.9 Hz, *J*_5b–5a_ = 11.0 Hz, H^5b^), 5.08 (dd, 1H, *J*_2–1_ = 3.6 Hz, *J*_2–3_ = 10.0 Hz, H^2^), 5.11 (ddd, 1H, *J*_4–5b_ = 5.9 Hz, *J*_4–5a_ = 7.5 Hz, *J*_4–3_ = 11.0 Hz, H^4^), 5.58 (dd, 1H, *J*_3–2_ = *J*_3–4_ = 10.0 Hz, H^3^), 6.50 (d, 1H, *J*_1–2_ = 3.6 Hz, H^1^), 8.67 [s, 1H, C(N*H*)CCl_3_] ppm. ^13^C NMR (CDCl_3_) *δ*: 20.5, 20.6, 20.7, 60.8, 68.6, 69.3, 69.9, 76.7, 77.0, 77.3, 93.1, 160.9, 169.8 ppm. IR (film): ν = 3481, 2500, 1757, 1487, 1218, 1046 cm^−1^. [α]^20^_D_ = +45.2 (c 0.2, CHCl_3_). The physico-chemical data are in agreement with those reported in the literature [22].

*Synthesis of* α*-allyl 3,5,6-tri-O-benzyl-(2,3,4-tri-O-acetyl-2-O-β-D-xylopyranosyl)-D-glucofuranoside* (**19a**). A mixture of **12a** (0.35 g, 0.71 mmol) and **14a** (0.358 g, 0.86 mmol) in dry CH_2_Cl_2_ (8.75 mL) under N_2_ atmosphere and in the presence of activated 4Å molecular sieves (0.8 g) was stirred for 30 min at room temperature, cooled at −20 °C and TMSOTf (6 mg, 5 μL, 0.03 mmol) was then added. The mixture was stirred again at room temperature for 6 h, quenched with Et_3_N, filtered on Celite^®^, and concentrated. Purification of the residue by column chromatography (petroleum ether/Et_2_O 6:4) gave **19a** as a colorless oil (*R*_f_ 0.4, petroleum ether/Et_2_O 1:1). Yield: 45% (0.24 g, 0.32 mmol). MS (ESI): 766 (M + NH_4_^+^). ^1^H NMR (CDCl_3_) *δ*: 1.99 (s, 3H, C*H*_3_CO), 2.03 (s, 3H, C*H*_3_CO), 2.08 (s, 3H, CH_3_CO), 3.37 (dd, 1H, *J*_5’b–4’_ = 7.0 Hz, *J*_5’b–5’a_ = 12.2 Hz, H^5’b^), 3.71 (dd, 1H, *J*_6b–5_ = 6.1 Hz, *J*_6b–6a_ = 10.5 Hz, H^6b^) 3.84 (dd, 1H, *J*_6a–5_ = 2.4 Hz, *J*_6a–6b_ = 10.5 Hz, H^6a^), 3.97–4.05 (m, 3H, H^2^, H^5^, H^7b^), 4.18–4.23 (m, 1H, H^7a^), 4.22 (dd, 1H, *J*_5’a–4’_ = 4.5 Hz, *J*_5’a–5’b_ = 12.2 Hz, H^5’a^), 4.28 (dd, 1H, *J*_3–2_ = 5.0 Hz, *J*_3–4_ = 6.5 Hz, H^3^), 4.33 (dd, 1H, *J*_4–3_ ≅ *J*_4–5_ = 6.5 Hz, H^4^), 4.51–4.58 (m, 5H, OC*H*_2_C_6_H_5_), 4.63 (d, 1H, *J*_1’–2’_ = 5.9 Hz, H^1’^) 4.80 (d, 1H, *J* = 11.6 Hz, OC*H*_2_C_6_H_5_), 4.93 (ddd, 1H, *J*_4’–5’a_ = 4.5 Hz, *J*_4’–3’_ ≅ *J*_4’–5’b_ = 7.0 Hz, H^4’^), 4.99 (dd, 1H, *J*_2’–1’_ = 5.9 Hz, *J*_2’–3’_ = 8.0 Hz, H^2’^), 5.02 (d, 1H, *J*_1–2_ = 4.3 Hz, H^1^), 5.12 (dd, 1H, *J*_3’–4’_ = 7.0 Hz, *J*_3’–2’_ = 8.0 Hz, H^3’^), 5.16 (dddd, 1H, *J*_9b–9a_ = *J*_9b–7a_ = *J*_9b–7b_ = 1.6 Hz, *J*_9b–8_ = 10.5 Hz, H^9b^ OCH_2_CHCH*H*), 5.30 (dddd, 1H, *J*_9a–9b_ = *J*_9a–7a_ = *J*_9a–7b_ = 1.6 Hz, *J*_9a–8_ = 17.2 Hz, H^9a^ OCH_2_CHC*H*H), 5.89 (dddd, 1H, *J*_8–7a_ = 5.1 Hz, *J*_8–7b_ = 6.1 Hz, *J*_8–9b_ = 10.5 Hz, *J*_8–9a_ = 17.2 Hz, H^8^ OCH_2_C*H*CH_2_), 7.26–7.33 (m, 15 H, ArH) ppm. ^13^C NMR (CDCl_3_) *δ*: 20.6, 20.7, 20.8, 61.7, 68.6, 68.8, 70.1, 70.6, 72.5, 72.6, 73.3, 76.0, 76.7, 76.8, 81.0, 84.9, 99.5, 100.7, 117.0, 122.4, 127.5, 127.6, 128.2, 128.4, 134.2, 137.8, 138.6, 138.8, 169.1, 169.9, 170.0 ppm. IR (CHCl_3_): ν = 3064, 1756, 1454, 1372, 1221, 1057 cm^−1^. [α]^20^_D_ = +17.6 (c = 0.2, MeOH).

*Synthesis of β-allyl 3,5,6-tri-O-benzyl-(2,3,4-tri-O-acetyl-2-O-β-D-xylopyranosyl)-D-glucofuranoside* (**19b**). A mixture of **12b** (0.35 g, 0.71 mmol) and **14a** (0.358 g, 0.86 mmol) in dry CH_2_Cl_2_ (8.75 mL) under N_2_ atmosphere and in the presence of activated 4Å molecular sieves (0.8 g) was stirred for 30 min at room temperature, cooled at −20 °C and TMSOTf (6 mg, 5 μL, 0.03 mmol) was then added. The mixture was stirred again at room temperature for 3.5 h, quenched with Et_3_N, filtered on Celite^®^, and concentrated. Purification of the residue by column chromatography (petroleum ether/Et_2_O 6:4) gave **19b** as a colorless oil (*R*_f_ 0.4, petroleum ether/Et_2_O 1:1). Yield: 60% (0.321 g, 0.43 mmol). MS (ESI): 766 (M + NH_4_^+^). ^1^H NMR (CDCl_3_) *δ*: 1.97 (s, 3H, CH_3_CO), 2.02 (s, 3H, CH_3_CO), 2.06 (s, 3H, CH_3_CO), 3.23 (dd, 1H, *J*_5’b–4’_ = 9.2 Hz, *J*_5’b–5’a_ = 12.0 Hz, H^5’b^), 3.68 (dd, 1H, *J*_6b–5_ = 5.2 Hz, *J*_6b–6a_ = 10.5 Hz, H^6b^), 3.88 (dd, 1H, *J*_6a–5_ = 2.0 Hz, *J*_6a–6b_ = 10.5 Hz, H^6a^), 3.94 (dd, 1H, *J*_3–2_ = 1.2 Hz, *J*_3–4_ = 5.0 Hz, H^3^), 3.97 (dddd, 1H, *J*_7b–9a_ = *J*_7b–9b_ = 1.5 Hz, *J*_7b–8_ = 6.0 Hz, *J*_7b–7a_ = 13.0 Hz, H^7b^, OCH*H*CHCH_2_), 4.06 (dd, 1H, *J*_5’a–4’_ = 5.2 Hz, *J*_5’a–5’b_ = 12.0 Hz, H^5’a^), 4.06 (ddd, 1H, J_5–6a_ = 2.0 Hz, *J*_5–6b_ = 5.2, *J*_5–4_ = 9.5 Hz, H^5^), 4.14 (dd, 1H, *J*_2–1_ = Hz, *J*_2–3_ = 1.2 Hz, H^2^), 4.19 (dd, 1H, *J*_4–3_ = 5.0 Hz, *J*_4–5_ = 9.5 Hz, H^4^), 4.19 (dddd, 1H, *J*_7a–9a_ = *J*_7a–9b_ = 1.5 Hz, *J*_7a–8_ = 5.0, *J*_7a–7b_ = 13.0 Hz, H^7a^ OC*H*HCHCH_2_), 4.24 (d, 1H, *J*_1’–2’_ = 7.2 Hz, H^1’^), 4.52 (d, 1H, *J* = 11.2 Hz, OC*H*_2_C_6_H_5_), 4.53 (d, 1H, *J* = 12.0 Hz, OC*H*_2_C_6_H_5_), 4.59 (s, 2H, OC*H_2_*C_6_H_5_), 4.60 (d, 1H, *J* = 12.0 Hz, OC*H*_2_C_6_H_5_), 4.79 (d, 1H, *J* = 11.2 Hz, OC*H*_2_C_6_H_5_), 4.83 (dd, 1H, *J*_2’–1’_ = 7.2 Hz, *J*_2’–3’_ = 8.5 Hz, H^2’^), 4.91 (ddd, 1H, *J*_4’–5’a_ = 5.2 Hz, *J*_4’–3’_ = 8.5 Hz, *J*_4’–5’b_ = 9.2 Hz, H^4’^), 5.01 (d, 1H, *J*_1–2_ = 1.2 Hz, H^1^), 5.08 (dd, 1H, *J*_3’–2’_ = *J*_3’–4’_ = 8.5 Hz, H^3’^), 5.18 (dddd, 1H, *J*_9b–9a_ = *J*_9b–9a_ = *J*_9b–7b_ = 1.5 Hz, *J*_9b–8_ = 10.5 Hz, H^9b^ OCH_2_CHCH*H*), 5.28 (dddd, 1H, *J*_9a–9b_ = *J*_9a–7a_ = *J*_9a–7b_ = 1.5 Hz, *J*_9a–8_ = 17.0 Hz, H^9a^ OCH_2_CHC*H*H), 5.89 (dddd, 1H, *J*_8–7a_ = 5.0 Hz, *J*_8–7b_ = 6.0 Hz, *J*_8–9b_ = 10.5 Hz, *J*_8–9a_ = 17.0 Hz, H^8^ OCH_2_C*H*CH_2_), 7.25–7.35 (m, 15 H, ArH) ppm. ^13^C NMR (MeOD) *δ*: 19.1, 19.1, 19.3, 61.9, 68.5, 68.8, 69.8, 70.9, 71.8, 71.8, 71.8, 73.0, 76.4, 79.9, 80.4, 84.6, 100.1, 106.6, 115.7, 117.1, 127.2, 127.4, 127.4, 127.5, 127.8, 127.9, 127.9, 128.1, 134.2, 137.9, 138.4, 138.7, 169.6, 170.0, 170.2 ppm. IR (CHCl_3_): ν = 3064, 1756, 1454, 1372, 1221, 1057 cm^−1^. [α]^20^_D_ = −12.8 (c 0.2, MeOH).

*Synthesis of β-allyl 3,5,6-tri-O-benzyl-2-O-β-D-xylopyranosyl-D-glucofuranoside* (**22b**). To a solution of **19b** (0.21 g, 0.29 mmol) in dry MeOH (62 mL) MeONa was added (0.038 g, 0.70 mmol) and the mixture was stirred at room temperature for 3 h, then concentrated. Purification of the residue by column chromatography (cyclohexane/EtOAc 1:9) gave **22b** as a pale-yellow oil (*R*_f_: 0.3, EtOAc). Yield: 90% (0.161 g, 0.26 mmol). MS (ESI): 605 (M + H^+^). ^1^H NMR (CDCl_3_) *δ*: 2.51 (br s, 1H, OH), 2.77 (br s, 1H, OH), 2.94 (br s, 1H, OH), 3.19 (dd, 1H, *J*_5’b-4’_ = 9.2, *J*_5’b-5’a_ = 12.0 Hz, H^5’b^), 3.29 (dd, 1H, *J*_2’-1’_ = 6.5 Hz, *J*_2’-3’_ = 8.0 Hz, H^2’^), 3.43 (dd, 1H, *J*_3’-2’_ = *J*_3’-4’_ = 8.0 Hz, H^3’^), 3.67 (ddd, *J*_4’-5’a_ = 4.8 Hz, *J*_4’-3’_ = 8.0 Hz, *J*_4’-5’b_ = 9.2 Hz, H^4’^), 3.70 (dd, 1H, *J*_6b-5_ = 4.5 Hz, *J*_6b–6a_ = 10.8 Hz, H^6b^), 3.88 (dd, 1H, *J*_6a–5_ = 2.0 Hz, *J*_6a–6b_ = 10.8 Hz, H^6a^), 3.95 (dd, 1H, *J*_5’a–4’_ = 4.8 Hz, *J*_5’a–5’b_ = 12.0 Hz, H^5’a^), 3.98 (dddd, 1H, *J*_7b–9a_ = *J*_7b–9b_ = 1.5 Hz, *J*_7b–8_ = 6.0 Hz, *J*_7b–7a_ = 13.0 Hz, H^7b^ OCH*H*CHCH_2_), 4.04 (dd, 1H, *J*_3–2_ = 1.0 Hz, *J*_3–4_ = 4.8 Hz, H^3^), 4.06 (ddd, 1H, J_5–6a_ = 2.0 Hz, *J*_5–6b_ = 4.5 Hz, *J*_5–4_ = 9.2 Hz, H^5^), 4.08 (d, 1H, *J*_1’–2’_ = 6.5 Hz, H^1’^), 4.19 (dddd, 1H, *J*_7a–9a_ = *J*_7a–9b_ = 1.5 Hz, *J*_7a–8_ = 5.0 Hz, *J*_7a–7b_ = 13.0 Hz, H^7a^ OC*H*HCHCH_2_), 4.23 (dd, 1H, *J*_2–1_ = *J*_2–3_ = 1.0 Hz, H^2^), 4.35 (dd, 1H, *J*_4–3_ = 4.8 Hz, *J*_4–5_ = 9.2 Hz, H^4^), 4.51 (d, 1H, *J* = 11.2 Hz, OC*H*_2_C_6_H_5_), 4.56 (d, 1H, *J* = 12.5 Hz, OC*H*_2_C_6_H_5_), 4.58 (d, 1H, *J* = 12.5 Hz, OC*H*_2_C_6_H_5_), 4.61 (d, 1H, *J* = 12.5 Hz, OC*H*_2_C_6_H_5_), 4.62 (d, 1H, *J* = 12.5 Hz, OC*H*_2_C_6_H_5_), 4.75 (d, 1H, *J* = 11.2 Hz, OC*H*_2_C_6_H_5_), 5.06 (d, 1H, *J*_1-2_ = 1.0 Hz, H^1^), 5.17 (dddd, 1H, *J*_9b–9a_ = *J*_9b–7a_ = *J*_9b–7b_ = 1.6 Hz, *J*_9b–8_ = 10.5 Hz, H^9b^ OCH_2_CHCH*H*), 5.28 (dddd, 1H, *J*_9a–9b_ = *J*_9a–7a_ = *J*_9a–7b_ = 1.6 Hz, *J*_9a–8_ = 17.0 Hz, H^9a^ OCH_2_CHC*H*H), 5.90 (dddd, 1H, *J*_8–7a_ = 5.0 Hz, *J*_8–7b_ = 6.0 Hz, *J*_8–9b_ = 10.5 Hz, *J*_8–9a_ = 17.0 Hz, H^8^ OCH_2_C*H*CH_2_), 7.28–7.37 (m, 15H, ArH) ppm. ^13^C NMR (CDCl_3_) *δ*: 64.7, 68.9, 69.4, 70.3, 72.4, 72.4, 72.4, 72.6, 73.4, 75.1, 79.9, 80.5, 84.6, 101.9, 106.6, 117.0, 127.5, 127.6, 127.7, 127.7, 127.9, 128.0, 128.3, 128.3, 128.4, 134.1, 137.7, 138.7, 138.7 ppm. IR (CHCl_3_): ν = 3415, 3019, 1785, 1450, 1368, 1045 cm^−1^. [α]^20^_D_ = –19.6 (c = 0.2, MeOH).

*Synthesis of 3,5,6-tri-O-benzyl-2-O-β-D-xylopyranosyl-α-D-glucofuranoside and 3,5,6-tri-O-benzyl-2-O-β-D-xylopyranosyl-β-D-glucofuranoside* (**23**). To a solution of **22b** (0.07 g, 0.12 mmol) in MeCOONa (0.034 g, 0.42 mmol) and MeCOOH (0.22 mL), PdCl_2_ (0.04 g, 0.22 mmol), and H_2_O (11 μL) were added. The mixture was stirred at room temperature for 12 h, filtered on Celite^®^, and washed with EtOAc. The filtrate was washed with a saturated NaHCO_3_ solution and extracted with EtOAc. The combined organic layers were dried over Na_2_SO_4_ and concentrated. Purification of the residue by column chromatography (EtOAc/cyclohexane 8:2) gave **23** (α/β 2:3) as a colorless oil (*R*_f_: **23a** 0.2, **23b** 0.3; EtOAc/MeOH 99:1). Yield: 42% (0.027 g, 0.05 mmol). MS (ESI): 600 (M + NH_4_^+^). ^1^H NMR (MeOD) *δ*: 3.11–3.21 (m, 3H), 3.23–3.30 (m, 3H), 3.44–3.54 (m, 3H), 3.66–3.73 (m, 2H), 3.82–3.91 (m, 3H), 3.93–3.98 (m, 2H), 4.08–4.11 (m, 1H), 4.11–4.14 (4H), 4.19 (dd, 1H, *J*_1_ = 2.6 Hz, *J*_2_ = 4.0 Hz), 4.25 (dd, 1H, *J*_1_ = 2.5 Hz, *J*_2_ = 4.6 Hz), 4.28 (d, 1H, *J* = 4.0 Hz), 4.30 (dd, 1H, *J*_1_ = *J*_2_ = 2.1 Hz), 4.38 (dd, 1H, *J*_1_ = 4.6 Hz, *J*_2_ = 7.9 Hz), 4.50–4.77 (m, 10H, OC*H*_2_C_6_H_5_), 5.25 (d, 1H, *J* = 1.0 Hz, H^1^ β-anomer), 5.39 (d, 1H, *J* = 4.0 Hz, H^1^ α-anomer), 7.26–7.38 (m, 30H, ArH) ppm. ^13^C NMR (MeOD) δ: 65.6, 65.6, 69.7, 69.7, 70.2, 70.2, 71.8, 71.9, 71.9, 71.9, 72.9, 73.0, 73.0, 73.3, 75.9, 76.4, 76.5, 76.9, 79.8, 80.7, 80.8, 81.6, 85.0, 85.0, 96.5, 102.3, 102.8, 103.0, 127.1, 127.1, 127.2, 127.3, 127.3, 127.4, 127.4, 127.4, 127.5, 127.7, 127.8, 127.8, 127.9, 127.9, 128.0, 128.0, 128.1, 128.1, 137.9, 138.0, 138.4, 138.4, 138.8, 138.8 ppm. IR (Nujol): ν = 3399, 1458, 1376, 1041 cm^−1^. [α]^20^_D_ = −32.6 (c = 0.3, MeOH).

*Synthesis of 2-O-β-D-xylopyranosyl-D-α-glucopyranose and 2-O-β-D-xylopyranosyl-D-β-glucopyranose* (**8**, sambubiose). To a solution of **23** (0.09 g, 0.15 mmol) in EtOH (9 mL), Pd/C 10% (0.018 g) was added and the mixture was hydrogenated under stirring at room temperature at 1 atm for 6 h, then filtered on Celite^®^, washed with EtOH, and kept overnight. The crystals obtained were then filtered and triturated to obtain **8** (sambubiose) as a white solid (*R*_f_: 0.2, CH_3_CN/H_2_O 9:1). Yield 51% (0.024 g, 0.08 mmol). Mp: 132–134 °C (petroleum ether). MS (ESI): 313 (M + 1), 311 (M-1). ^1^H NMR (DMSO*-*d^6^) *δ*: 3.02–3.05 (m, 2H, H^5^, H^2’^), 3.08–3.17 (m, 3H, H^2^, H^3’^, H^5’a^), 3.23–3.28 (m, 1H, H^4’^), 3.41–3.47 (m, 1H, H^6a^), 3.54–3.68 (m, 4H, H^3^, H^4^, H^6b^, H^5’b^), 4.25 (d, 1H, *J*_1’–2’_ = *7*.2 Hz, H^1’^), 4.39 (dd, 1H, *J*_OH6–6a_ ≅ *J*_OH6–6b_ = 5.5 Hz, OH^6^), 4.77 (d, 1H, *J* = 3.2 Hz, OH), 4.93 (d, 1H, *J* = 5.6 Hz, OH), 4.98 (d, 1H, *J* = 5.2 Hz, OH^4’^), 5.00 (d, 1 H, *J* = 4.8 Hz, OH), 5.02–5.05 (m, 2H, H^1^, OH) 6.34 (d, 1H, *J* = 4.5 Hz, OH^1^) ppm. ^13^C NMR (DMSO-d^6^) *δ*: 61.5 (C6), 66.1 (C5’), 69.9 (C4’), 70.5 (C5), 72.1 (C3), 72.4 (C4), 74.2 (C2’), 76.7 (C3’), 82.7 (C2), 92.0 (C1), 106.4 (C1’). α-anomer ^13^C NMR (D_2_O) *δ*: 60.4 (C6), 65.0 (C5’), 69.2 (C4’), 69.4 (C5), 71.0 (C3), 71.7 (C4), 73.1 (C2’), 75.5 (C3’), 80.7 (C2), 91.7 (C1), 104.7 (C1’). β-anomer ^13^C NMR (D_2_O) *δ*: 60.6 (C6), 65.1 (C5’), 69.1 (C4’), 69.4 (C5), 73.4 (C2’), 75.5, 75.69, 75.64, 81.8 (C2), 94.6 (C1), 103.6 (C1’). IR (Nujol): ν = 3400, 2840, 1070 cm^−1^. [α]^20^_D_ = −5.0 (c = 0.03, MeOH). [10]

## 4. Conclusions

An efficient method for the total synthesis of natural disaccharide sambubiose was developed. Our investigation enabled us to identify the best combination of donor and acceptor glycosides as well as a catalyst that allowed a complete stereocontrol of the key *O*-glycosylation step. The protocol described herein was carried out using stable, safe, non-toxic agents and a step-by-step economical synthetic route. At the same time, a satisfactory overall yield and the characterization of sambubiose were achieved, thus overcoming the drawbacks associated with the current method reported in the literature [10].

## Figures and Tables

**Table 1:** Optimization of the *O*-glycosylation reaction.

Entry	Donor	Acceptor	Catalyst	Reaction Time (h)	Product	Yield (%)
1	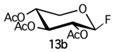	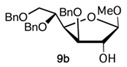	BF_3_^.^ OEt_2_	4	**18b**	80
2	**13b**	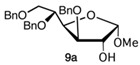	BF_3_^.^ OEt_2_	4	**18a**	50
3	**13b**	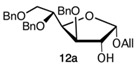	BF_3_^.^ OEt_2_	6	**19a**	-
4	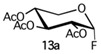	**9b**	BF_3_^.^ OEt_2_	6	**18b**	-
5	**13a**	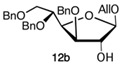	BF_3_^.^ OEt_2_	6	**19b**	-
6	**13b**	**12b**	BF_3_^.^ OEt_2_	6	**19b**	30
7	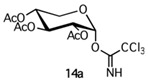	**9b**	BF_3_^.^ OEt_2_	12	**18b**	44
8	**14a**	**12b**	TMSOTf	3.5	**19b**	60
9	**14a**	**12a**	TMSOTf	6	**19a**	45
